# Defunctionalisation catalysed by boron Lewis acids

**DOI:** 10.1039/d0sc03712e

**Published:** 2020-07-28

**Authors:** Huaquan Fang, Martin Oestreich

**Affiliations:** Institut für Chemie, Technische Universität Berlin Strasse des 17. Juni 115 10623 Berlin Germany martin.oestreich@tu-berlin.de

## Abstract

Selective defunctionalisation of organic molecules to valuable intermediates is a fundamentally important transformation in organic synthesis. Despite the advances made in efficient and selective defunctionalisation using transition-metal catalysis, the cost, toxicity, and non-renewable properties limit its application in industrial manufacturing processes. In this regard, boron Lewis acid catalysis has emerged as a powerful tool for the cleavage of carbon–heteroatom bonds. The ground-breaking finding is that the strong boron Lewis acid B(C_6_F_5_)_3_ can activate Si–H bonds through η^1^ coordination, and this Lewis adduct is a key intermediate that enables various reduction processes. This system can be tuned by variation of the electronic and structural properties of the borane catalyst, and together with different hydride sources high chemoselectivity can be achieved. This Perspective provides a comprehensive summary of various defunctionalisation reactions such as deoxygenation, decarbonylation, desulfurisation, deamination, and dehalogenation, all of which catalysed by boron Lewis acids.

## Abstract 2

The combination of boron Lewis acid catalysts and hydride sources enables the cleavage of various carbon–heteroatom bonds.

## Graphical abstract

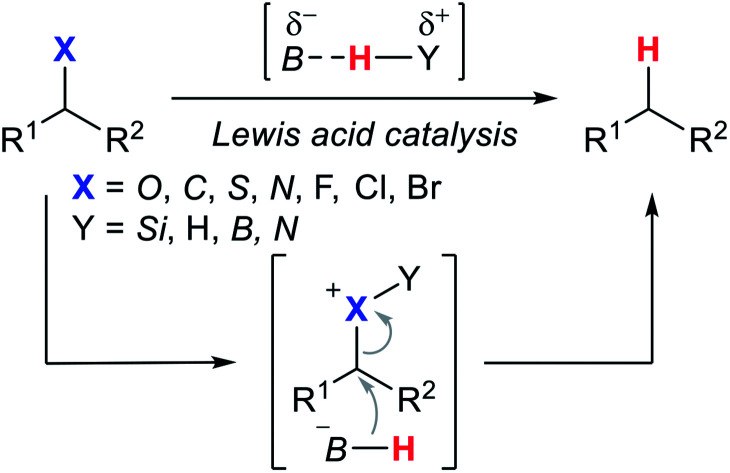

## Introduction

The conversion of organic functional groups into hydrogen atoms, namely defunctionalisation, is an important transformation in synthetic chemistry. Although it turns more functionalised raw materials into less functionalised products, the latter are considered to be more valuable than their precursors for diverse aspects such as high-value feedstocks produced by degradation of biomass sources^1^ and environmentally friendly fuels prepared by desulfurisation and deamination of crude liquid fuels.^2^ In addition, this process has also found application in environmental remediation, for example, dechlorination of toxic persistent polychlorinated biphenyls (PCBs).^3^

**Scheme 1:**
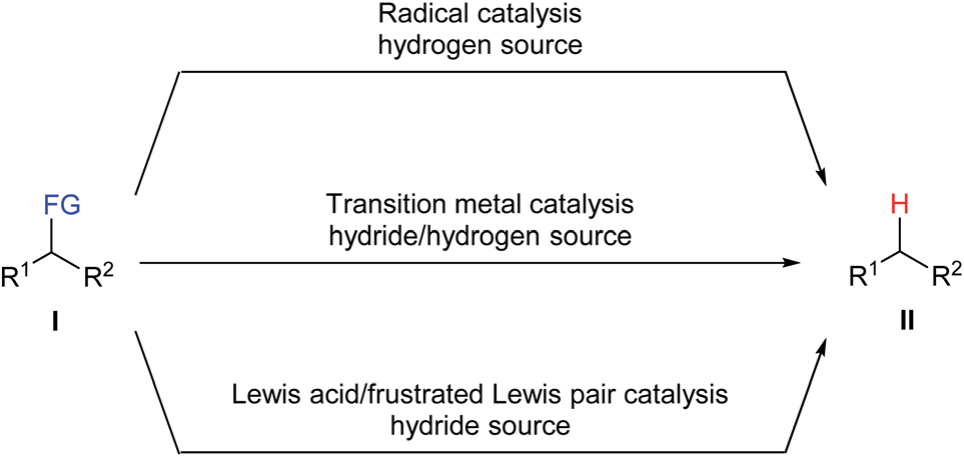
Ways of catalytic defunctionalisation. FG = functional group.

Numerous methods have been developed for the defunctionalisation of a variety of functional groups. Traditional approaches generally utilise stoichiometric amounts of pyrophoric metallic hydrides as reductant. Although widely used in laboratories, this approach is only applicable to certain leaving groups and suffers from the formation of inorganic salts as stoichiometric waste as well as poor selectivity and functional-group tolerance. Catalytic approaches are highly demanded and can serve as convenient, efficient, and economic alternatives for established methods. Three common catalytic strategies for the defunctionalisation of a variety of functional groups, including radical catalysis, transition-metal catalysis, and Lewis acid/frustrated Lewis pair catalysis, have been developed (Scheme 1). Reductive radical chain defunctionalisation of **I** with tin hydrides as the hydrogen source in the presence of a radical starter had been developed over the last 60 years.^4^ However, this method suffers from the use of toxic organotin compounds and the difficulty to completely remove the corresponding tin by-products. Several improvements including the use of a catalytic amount of tin hydrides and the use of other hydrogen sources have also been developed. Transition metal-catalysed defunctionalisation of **I** with a hydrogen source has provided an efficient and selective protocol for the cleavage of carbon–heteroatom bonds.^5^ However, the use of rare, expensive, and toxic transition metal catalysts limit their applications in industrial manufacturing processes. Recently, Lewis acid/frustrated Lewis pair-catalysed defunctionalisation of **I** with various hydride sources has emerged as a promising tool to this end.^6^

**Scheme 2:**
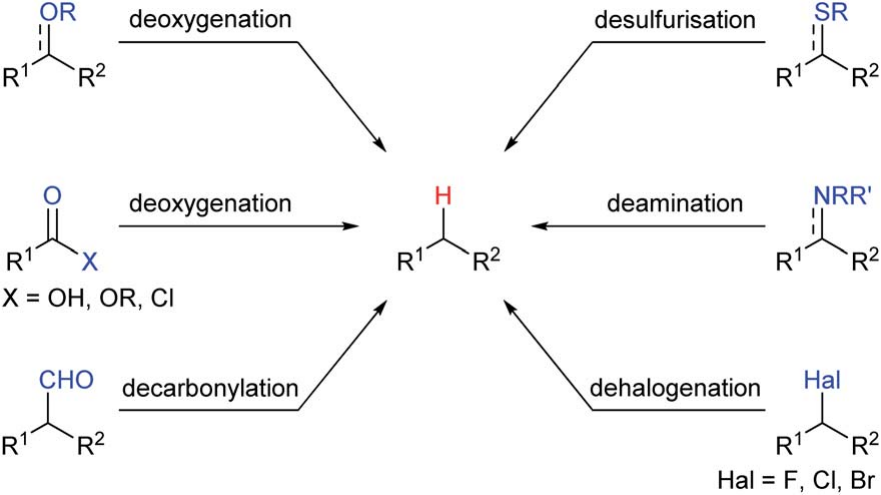
Scope of boron Lewis acids-catalysed defunctionalisation.

Among various Lewis acids investigated, boron Lewis acids are particularly attractive due to their high Lewis acid strength, low cost, and benign environmental impact and have been developed rapidly in the last two decades. What's more, the Lewis acidity and reactivity of boron Lewis acid can be easily tuned by changing or modifying substituents attached to the boron atom.^7^ A variety of defunctionalisation reactions catalysed by boron Lewis acids, such as deoxygenation, decarbonylation, desulfurisation, deamination, and dehalogenation, have been reported (Scheme 2). This review provides a comprehensive summary of defunctionalisation catalysed by boron Lewis acids. The condensation of alkoxysilanes and hydrosilanes to synthesize structurally complex functional silicones and an alkane as a by-product catalysed by a B(C_6_F_5_)_3_ catalyst, known as the Piers–Rubinsztajn reaction,^8^ is not covered.

## Boron Lewis acids-catalysed deoxygenation

**Scheme 3:**
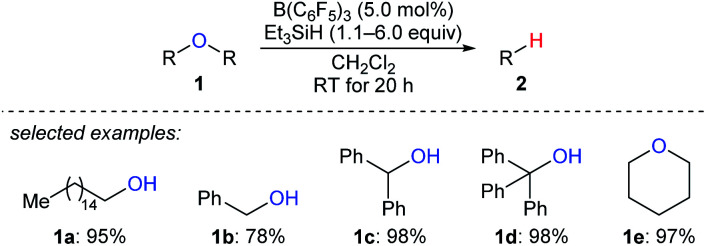
Deoxygenation of alcohols and ethers catalysed by B(C_6_F_5_)_3_ with Et_3_SiH as the reductant.

Deoxygenation of organic molecules to hydrocarbons is a step frequently encountered in organic synthesis. In fact, boron Lewis acid-mediated deoxygenation of alcohols and their derivatives with a hydrosilane as the reductant is known since the 1970s.^9^ However, the application of boron Lewis acid in catalysis remained undeveloped until 1999. Inspired by the pioneering work of Piers *et al.* on B(C_6_F_5_)_3_-catalysed hydrosilylation of carbonyl functions,^10^ Gevorgyan, Yamamoto, and co-workers found that alcohols and ethers were effectively reduced by excess Et_3_SiH in the presence of catalytic amounts of B(C_6_F_5_)_3_ to give the corresponding hydrocarbons at room temperature (Scheme 3).^11^ This catalytic system is efficient for the deoxygenation of primary alcohols, however, the deoxygenation of secondary and tertiary alcohols, except for those alcohols possessing strong carbocation-stabilising groups, was failed. Hence, the relative reactivity order of different types of alcohols was found to be 1° ≫ 2° > 3°.

**Scheme 4:**
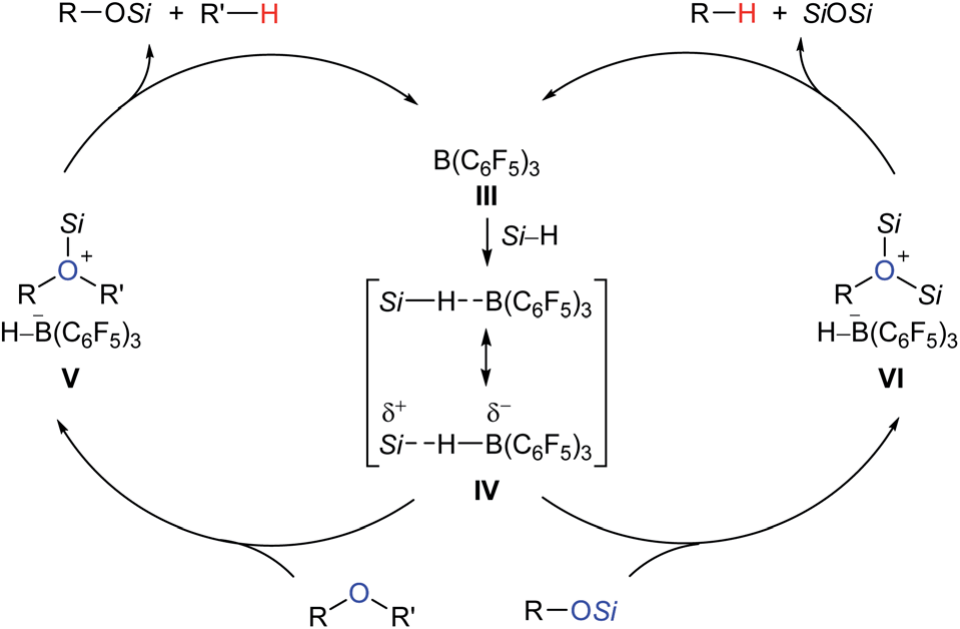
Proposed mechanism for B(C_6_F_5_)_3_-catalysed deoxygenation of alcohols and ethers.

A proposed mechanism for B(C_6_F_5_)_3_-catalysed deoxygenation of alcohols and ethers is depicted in Scheme 4.^11*b*,12^ The association of B(C_6_F_5_)_3_ with hydrosilane generates an η^1^-adduct **IV**, either represented as *Si*–H⋯B(C_6_F_5_)_3_ or *Si*⋯H–B(C_6_F_5_)_3_. The adduct is subsequently attacked by the substrate to form ion pairs **V** or **VI**; hydride transfer from the borohydride to the electrophilic carbon atom of the oxonium ion produces the silyl ether and hydrocarbon, respectively, with regeneration of **III**. It is worth noting that Lewis adduct **IV** is usually spectroscopically undetectable when mixing B(C_6_F_5_)_3_ and a hydrosilane. However, an isolable borane–hydrosilane adduct formed by mixing 1,2,3-tris(pentafluorophenyl)-4,5,6,7-tetrafluoro-1-boraindene and Et_3_SiH was reported by Piers, Tuononen, and co-workers.^13^

**Scheme 5:**
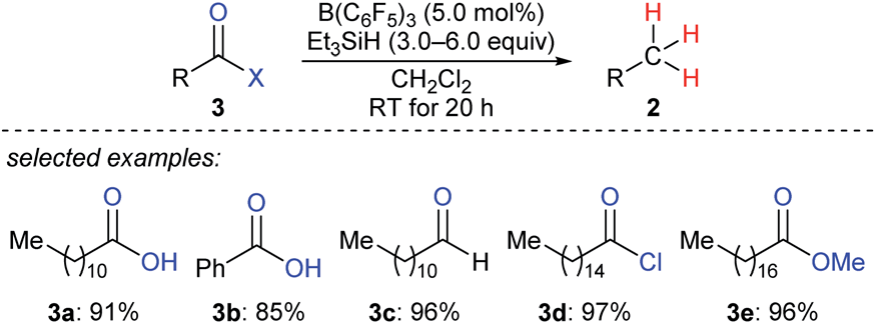
Deoxygenation of carbonyl and carboxy compounds catalysed by B(C_6_F_5_)_3_ with Et_3_SiH as the reductant.

By using that catalytic system, Gevorgyan, Yamamoto, and co-workers described the exhaustive deoxygenation of a variety of carbonyl functions such as carboxylic acids, aldehydes, acyl chlorides, and esters to give the corresponding hydrocarbons at room temperature (Scheme 5).^14^

**Scheme 6:**
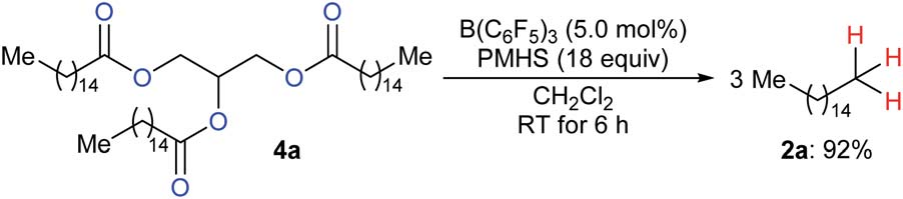
Deoxygenation of biomass-derived fatty acid esters catalysed by B(C_6_F_5_)_3_ with PMHS as the reductant.

To produce long-chain hydrocarbons (carbon number > 10), Fu and co-workers developed a B(C_6_F_5_)_3_-catalysed deoxygenation of biomass-derived fatty acids and derivatives thereof with the silicone industry byproduct polymethylhydrosiloxane (PMHS) as the reductant (Scheme 6).^15^ The successful conversion of commercially available plant oils to hydrocarbons demonstrated the value of this method and also provided a useful strategy for the production of liquid hydrocarbon fuels by upgrading of biodiesel. Later, B(C_6_F_5_)_3_-catalysed deoxygenation of triglycerides to give a mixture of alkanes and alkenes with hydrosiloxanes as reductants was further explored by Gale and Brook (not shown).^16^

**Scheme 7:**
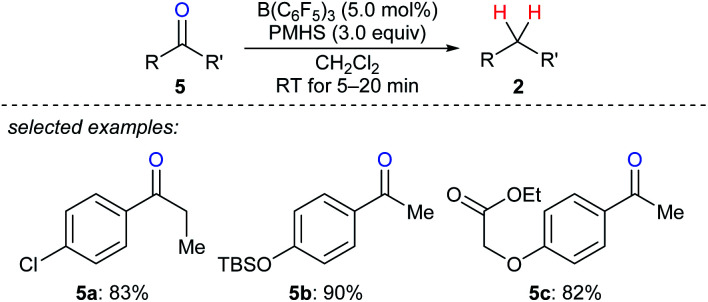
Deoxygenation of ketones catalysed by B(C_6_F_5_)_3_ with PMHS as the reductant.

A mild and rapid B(C_6_F_5_)_3_-catalysed deoxygenation of a variety of ketones **5a–c** to afford the corresponding hydrocarbons with PMHS as the reductant was disclosed by Chandrasekhar and co-workers (Scheme 7).^17^ This system exhibits good efficiency and selectivity and is compatible with various functional groups such as chloride, silyl ether, ester, and alkenyl groups. Later, these authors employed this catalytic system for the deoxygenation of Baylis–Hillman adducts to form (*Z*)- or (*E*)-trisubstituted alkenes (not shown);^18^ the process involves the elimination of the hydroxy group followed by double bond migration. In addition, Cantat and co-workers developed B(C_6_F_5_)_3_-catalysed deoxygenation of oxalic acid with tetramethyldisiloxane (TMDS) or PMHS as reductants (not shown).^19^ Nimmagadda and McRae found the combined use of B(C_6_F_5_)_3_ with the less sterically hindered Et_2_SiH_2_ or *n*BuSiH_3_ proved to be a more reactive catalytic system for the deoxygenation of polycarboxylic acids, aldehydes, ketones, and alcohols (not shown).^20^ The B(C_6_F_5_)_3_/Ph_2_SiH_2_ catalytic system demonstrated by Tan and Zhang was shown to be efficient for the reduction of an alcohol, a ketone, an α,β-unsaturated carboxylic acid, and an enol ether to their corresponding hydrocarbons (not shown).^21^

**Scheme 8:**
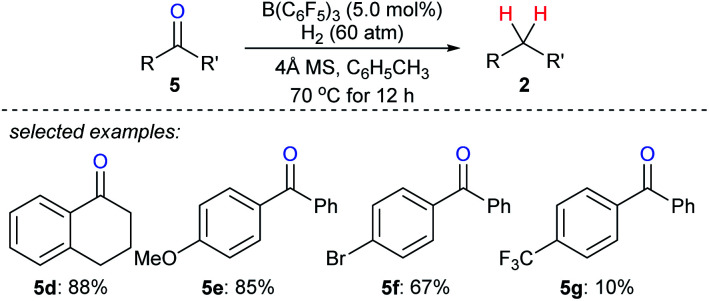
Deoxygenation of ketones catalysed by B(C_6_F_5_)_3_ with H_2_ as the reductant.

The use of H_2_ as a reductant in boron Lewis acid-catalysed deoxygenation is challenging yet highly attractive, and only water is formed as a by-product. Repo and co-workers found that Lewis pair of B(C_6_F_5_)_3_ and aromatic carbonyl compounds can heterolytically activate H_2_ at elevated temperature (110 °C).^22^ By this, the stoichiometric reduction of benzophenone with H_2_ as the reductant became feasible. However, the catalytic deoxygenation of ketones with H_2_ by B(C_6_F_5_)_3_ still is a difficult task due to the hydrolysis of B(C_6_F_5_)_3_ with the by-product H_2_O. By employing molecular sieves as a heterogeneous Lewis base and a desiccant to adsorb water, Mahdi and Stephan developed a B(C_6_F_5_)_3_-catalysed deoxygenation of diaryl ketones **5d–g** with H_2_ as the reductant at 70 °C (Scheme 8).^23^

**Scheme 9:**
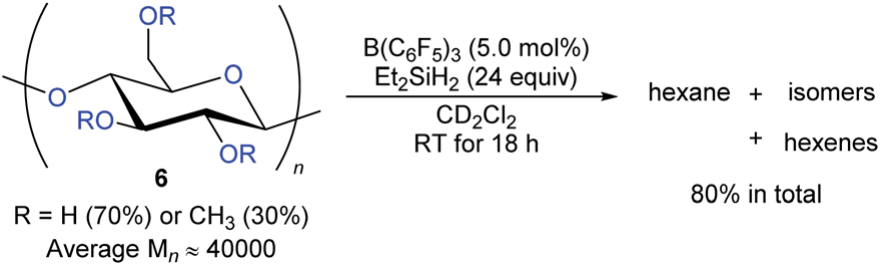
Deoxygenation of carbohydrates catalysed by B(C_6_F_5_)_3_ with Et_2_SiH_2_ as the reductant.

The degradation of readily available carbohydrates to value-added feedstocks and fuels is an attractive yet challenging endeavour which requires the activation of several nonactivated C–O bonds. In 2014, Gagné and co-workers reported the B(C_6_F_5_)_3_-catalysed deoxygenation of carbohydrates **6** to afford mixtures of hexanes and hexenes with Et_2_SiH_2_ as the reductant (Scheme 9).^24^ The degree of deoxygenation was influenced by the choice of hydrosilane. Secondary hydrosilanes as reductants led to exhaustive reduction while tertiary hydrosilanes generated partially deoxygenated products. Later, B(C_6_F_5_)_3_-catalysed deoxygenation of lignin^25^ and various polymeric materials based on polyethers, polyesters, polycarbonates,^26^ and poly(methyl acrylate)^27^ with hydrosilanes as reductants were demonstrated by the groups of Cantat, Chang, and Seo (not shown).

**Scheme 10:**
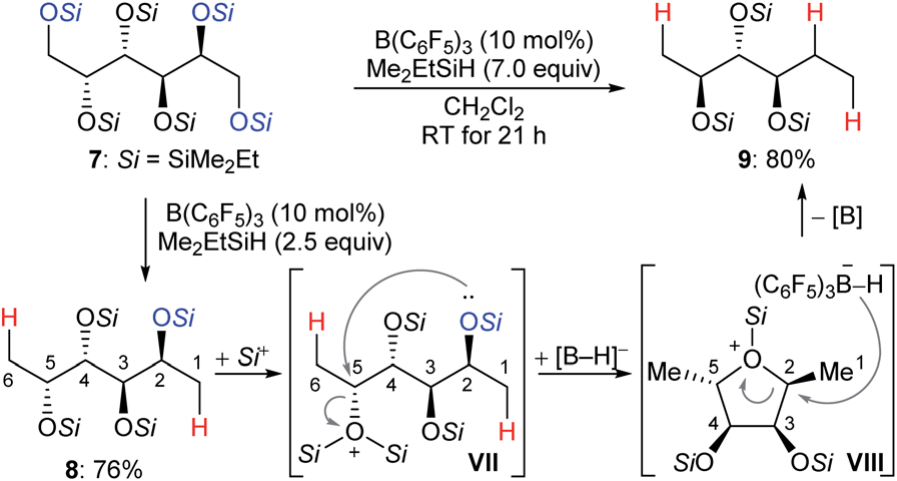
Selective deoxygenation of biologically sourced polyols catalysed by B(C_6_F_5_)_3_ with Me_2_EtSiH as the reductant.

The chemoselective partial deoxygenation of a variety of biologically sourced polyols to produce various oxygen-functionalised chiral synthons using a combination of B(C_6_F_5_)_3_ and tertiary hydrosilanes was described by Gagné and co-workers (Scheme 10).^28^ For example, the deoxygenation of galactitol **7** with Me_2_EtSiH (7.0 equiv.) in the presence of B(C_6_F_5_)_3_ (10 mol%) generated a C2-reduced triol **9** with inversion at C5. The deoxygenation of **7** with 2.5 equivalents of Me_2_EtSiH gave 1,6-deoxygenated tetraol **8**, which underwent intramolecular nucleophilic attack from the C2–OSi group to the activated C5–OSi_2_^+^ in **VII** to generate the cyclic oxonium ion intermediate **VIII**. Subsequent hydride transfer from borohydride to the C2 position of **VIII** formed the observed triol **9** with inversion at C5. The formation of cyclic intermediates caused by neighbouring group participation is crucial for achieving high site- and chemoselectivity. Later, these authors reported B(C_6_F_5_)_3_-catalysed chemoselective deoxygenation of unsaturated polyols to produce highly enriched (*Z*)-triols and partial deoxygenation of disaccharides to yield 1,6-deoxygenated tetraols and 1-deoxyglucose with a tertiary hydrosilane as the reductant (not shown).^29^ Moreover, site- and chemoselective deoxygenations of carbohydrates and its derivatives by a combination of B(C_6_F_5_)_3_/catecholborane (HBcat) and B(3,5-(CF_3_)_2_C_6_H_3_)_3_/tertiary hydrosilane combinations were also developed (not shown).^30^ More recently, the Gagné group found the polarity of solvent to exert a profound influence on reactivity and regioselectivity of the deoxygenation of sugars using the B(C_6_F_5_)_3_/hydrosilane catalytic system (not shown).^31^ Mechanistic investigations indicated low-dielectric solvents can shorten inter-ion bond lengths of the key ion-pair intermediates due to electrostatic compressive forces.

**Scheme 11:**
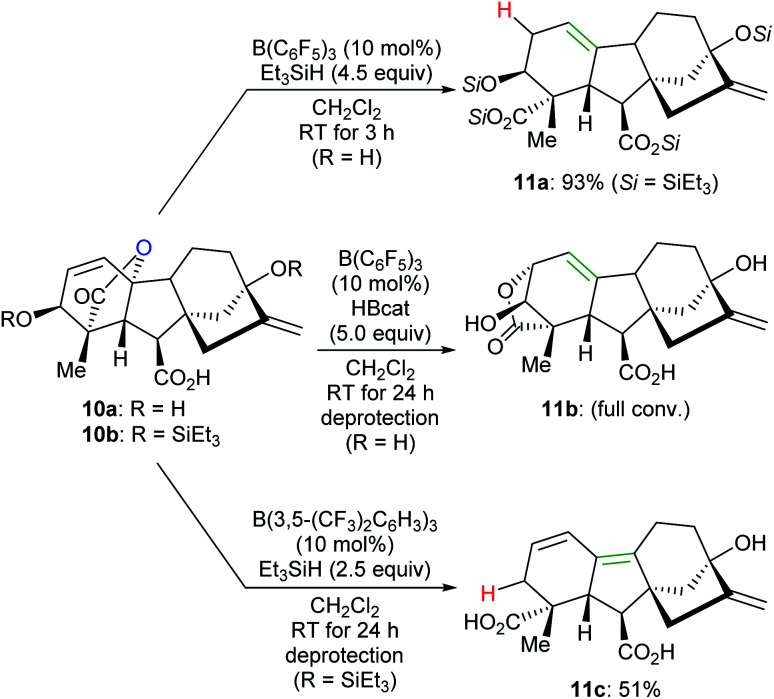
Chemoselective deoxygenation of gibberellic acids catalysed by B(C_6_F_5_)_3_ or B(3,5-(CF_3_)_2_C_6_H_3_)_3_ with Et_3_SiH or HBcat as reductants.

By tuning the electronic properties of fluoroaryl borane catalysts and utilising different reductants, chemo- and site-selective modifications of various complex natural products to yield divergent products were achieved by Gagné and co-workers (Scheme 11).^32^ For example, the reaction of gibberellic acid (**10a**) with excess Et_3_SiH in the presence of a catalytic amount of B(C_6_F_5_)_3_ generated the known diacid **11a** in 93% yield. This process involves a sequence of dehydrosilylation and ring-opening of the lactone group accompanied by an allylic transposition. In addition, **11a** can also be obtained in excellent yields by the deoxygenation of pre-silylated gibberellic acid **10b** with a combination of B(C_6_F_5_)_3_/HBcat or B(2,4,6-F_3_C_6_H_2_)_3_/Me_2_EtSiH (not shown). By employing a combination of B(C_6_F_5_)_3_/HBcat, full isomerisation of **10a** to **11b** was observed after deprotection. The deoxygenation of **10b** with excess Et_3_SiH catalysed by B(3,5-(CF_3_)_2_C_6_H_3_)_3_ provided a conjugated diene derivative of **11c** in 51% yield after deprotection.

**Scheme 12:**
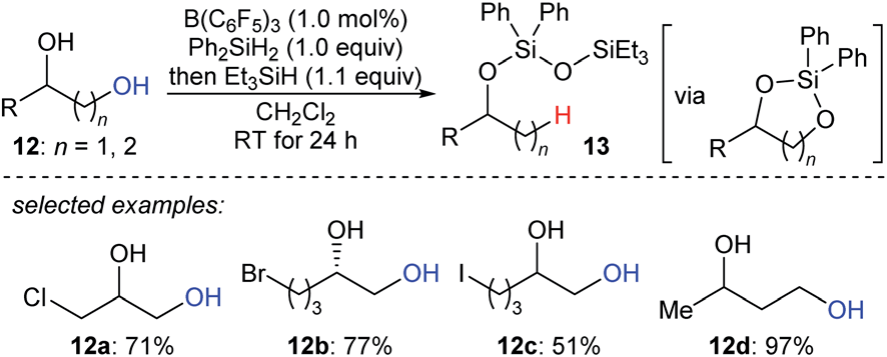
Selective deoxygenation of terminal 1,2- and 1,3-diols catalysed by B(C_6_F_5_)_3_ with Et_3_SiH as the reductant.

The beautiful work of Gagné prompted chemists to develop new approaches to selective deoxygenation. In 2015, Drosos and Morandi introduced a highly selective B(C_6_F_5_)_3_-catalysed monodeoxygenation of terminal 1,2- and 1,3-diols **12a–d** to give 2-alkanols by using a combination of Ph_2_SiH_2_ and Et_3_SiH (Scheme 12).^33^ The overall reaction is a sequence of protection to form cyclic siloxane intermediates and selective reduction at their primary position to afford 2-alkanols. Computational studies reveals that the formation of cyclic siloxane intermediates, which facilitates the deoxygenation by minimizing the steric repulsions between cyclic siloxane and borane–hydrosilane complex and hinders the further deoxygenation due to the bulky disiloxane moiety, plays a significant role.^34^

**Scheme 13:**
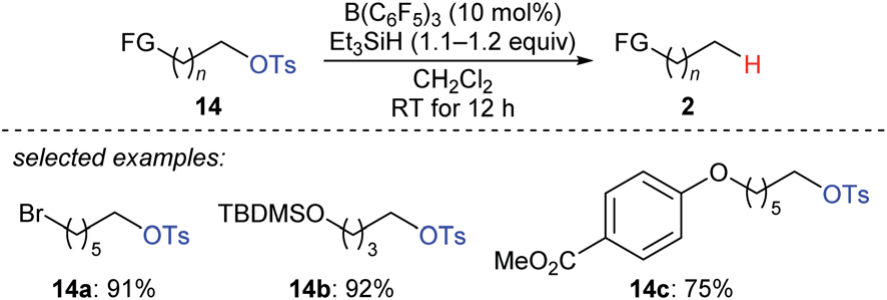
Selective deoxygenation of primary alkyl tosylates catalysed by B(C_6_F_5_)_3_ with Et_3_SiH as the reductant.

A two-step strategy for the B(C_6_F_5_)_3_-catalysed chemoselective deoxygenation of 1,*n*-diols and the hydroxymethyl group of an orthogonally protected carbohydrate with Et_3_SiH as the reductant was disclosed by Oestreich and co-workers (Scheme 13).^35^ The cleavage of C–O bonds of primary tosylates **14a–c** proceeds preferentially over that of bromide, silyl ethers, and aryl ethers at room temperature. Later, Song and co-workers used (HMe_2_SiCH_2_)_2_ as a new reductant for the chemoselective deoxygenation of ether-substituted alcohols and carbonyl compounds (not shown), and the authors proposed that (HMe_2_SiCH_2_)_2_ promotes an intramolecular Si–O activation pathway.^36^

**Scheme 14:**
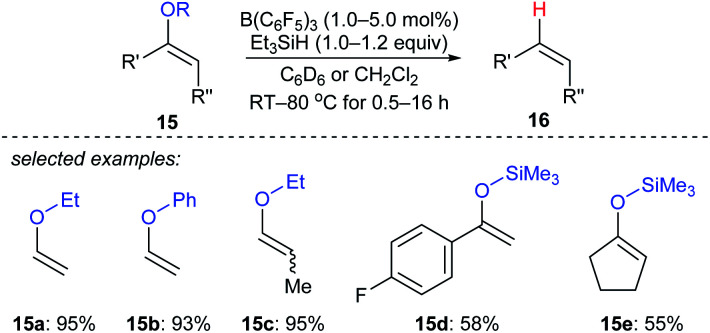
Selective deoxygenation of enol ethers catalysed by B(C_6_F_5_)_3_ with Et_3_SiH as the reductant.

Selective deoxygenation of enol ethers **15a–e** with Et_3_SiH as the reductant catalysed by B(C_6_F_5_)_3_ was achieved by Chulsky and Dobrovetsky (Scheme 14).^37^ This process involves the selective “indirect” cleavage of alkenyl–oxygen bonds in the presence of alkyl–oxygen bonds; the mechanism is believed to be a sequence of hydrosilylation followed by silicon-assisted β-elimination.

**Scheme 15:**
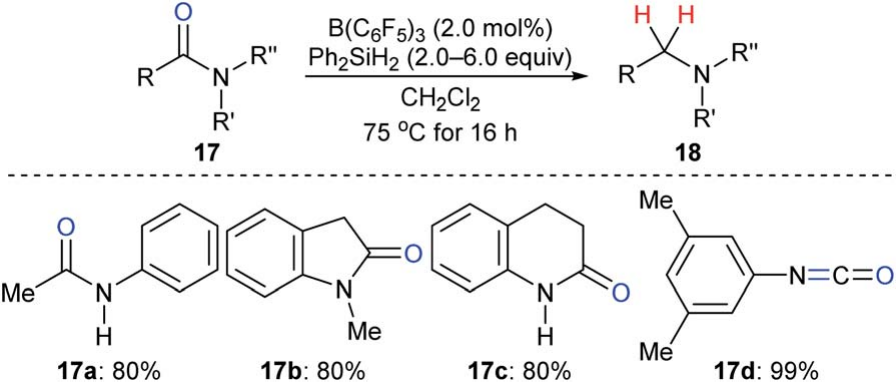
Deoxygenation of amides and isocyanate catalysed by B(C_6_F_5_)_3_ with Ph_2_SiH_2_ as the reductant.

The reduction of amides to the corresponding amines, which is another synthetically useful transformation in organic synthesis,^38^ with hydrosilanes as reductants catalysed by a boron Lewis acid was first described by Tan and Zhang (Scheme 15).^21^ Various *N*-phenylamides **17a–c** were successfully reduced to the corresponding amines at 75 °C. However, the reduction of the parent benzamide using this catalytic system was unsuccessful, even at 120 °C. Later, McGrath and co-workers found that various functional groups such as ether, ketone, and ester groups were tolerated in the B(C_6_F_5_)_3_-catalysed reduction of acetanilides to secondary amines with Et_3_SiH as the reductant (not shown).^39^ The reactivity of hydrosilanes with different steric demand in this reaction was also examined.

**Scheme 16:**
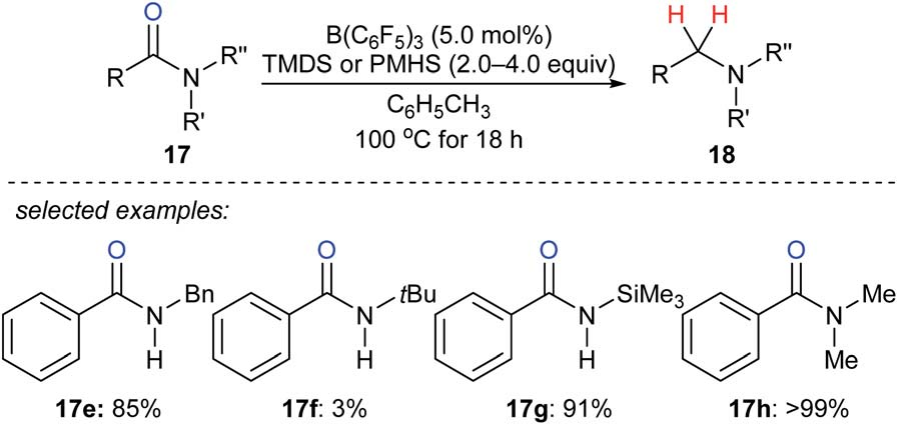
Deoxygenation of amides catalysed by B(C_6_F_5_)_3_ with TMDS or PMHS as reductants.

By utilising TMDS or PMHS as reductants, reduction of various secondary and tertiary amides **17e–h** to the corresponding amines catalysed by B(C_6_F_5_)_3_ were independently described by the groups of Cantat^40^ and Adronov^41^ (Scheme 16). The reduction of benzamide with TMDS (2.0 equiv.) in the presence of B(C_6_F_5_)_3_ (10 mol%) gave mixtures of dibenzylamine, *N*-benzylbenzamide, and (*E*)-*N*-benzyl-1-phenylmethanimine at 100 °C after 18 h. To prevent the formation of benzonitrile, which was formed by slow dehydrogenative silylation of the N–H bonds of benzamide and subsequent elimination of a siloxane, the protection of benzamide using Me_3_SiCl prior to the reduction was performed. Using this strategy, primary amides were successfully converted into the corresponding primary amines in excellent yields.

By merging Tf_2_O/2-F-pyridine activation and B(C_6_F_5_)_3_/TMDS reduction, Huang and co-workers found that various *N*-alkyl secondary amides, which had been difficult to reduce previously, were efficiently reduced to secondary amines at room temperature (not shown).^42^ A variety of functional groups such as methoxy, trifluoromethyl, bromo, nitro, ester, cyano, alkenyl, alkynyl, cyclopropyl, and silyl ether was compatible.

**Scheme 17:**
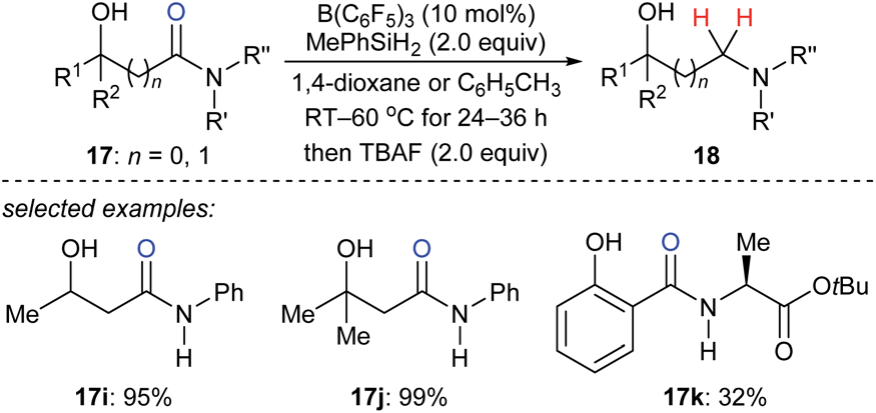
Hydroxy-directed deoxygenation of amides catalysed by B(C_6_F_5_)_3_ with MePhSiH_2_ as the reductant.

In 2018, Sohma, Kanai, and co-workers described a B(C_6_F_5_)_3_-catalysed chemo- and regioselective reduction of various hydroxy amides **17i–k** with MePhSiH_2_ as the reductant to synthesize 1,*n*-aminoalcohols under mild conditions with high functional group tolerance (Scheme 17).^43^ This process undergoes a sequence of B(C_6_F_5_)_3_-catalysed dehydrogenative silylation of the hydroxy group and selective deoxygenation through intramolecular Lewis acid/base type interaction between the silicon atom and oxygen atom of the amide carbonyl group. The application of this catalytic system to chemo- and site-selective reduction of a specific amide bond in cyclosporin A, which contains four secondary and seven tertiary amide bonds, demonstrated the power of this catalytic system when applied to complex molecules.

**Scheme 18:**
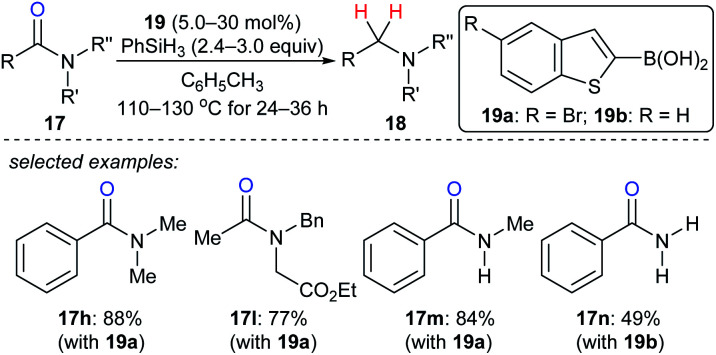
Deoxygenation of amides catalysed by boronic acids **19** with PhSiH_3_ as the reductant.

As described above, the strong boron Lewis acid B(C_6_F_5_)_3_ proved to be a potent catalyst in the reduction of amides. In 2013, Beller and co-workers introduced benzothiophene-functionalised boronic acids **19** for the reduction of tertiary, secondary, and primary amides with PhSiH_3_ as the reductant. At 110–130 °C, the corresponding amines were obtained and the functional-group tolerance was good (Scheme 18).^44^ Later, Blanchet and co-workers reported bis(2-chlorophenyl)borinic acid as an efficient catalyst for the reduction of tertiary amides with PhSiH_3_ under mild reaction conditions (not shown).^45^ Mechanistic investigations indicated that this process involves the formation of borane and an amine–borane complex.

**Scheme 19:**
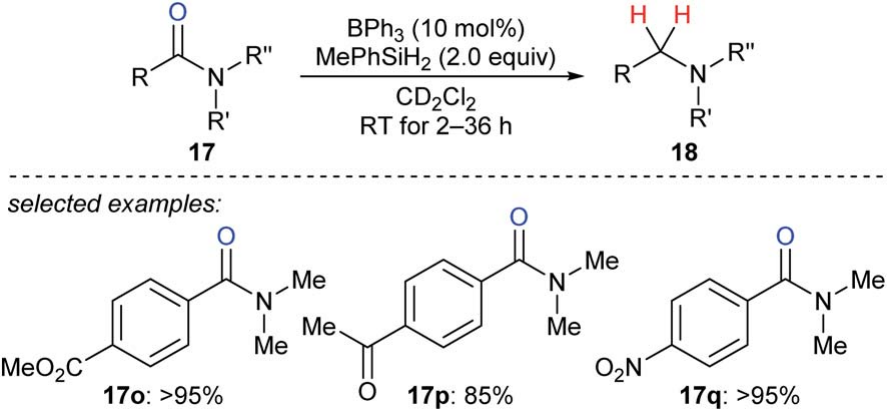
Deoxygenation of amides catalysed by BPh_3_ with MePhSiH_2_ as the reductant.

In 2016, Okuda and co-workers described the reduction of tertiary amides **17o–q** with MePhSiH_2_ as the reductant catalysed by moderately Lewis acidic BPh_3_ to give amines under mild conditions with high chemoselectivity in the presence of halide, nitro, ether, ketone, ester, imine, and isocyanate functions (Scheme 19).^46^ The authors proposed a carbonyl activation pathway for this catalytic system instead of the known Piers–Oestreich-type hydrosilane activation mechanism.

**Scheme 20:**
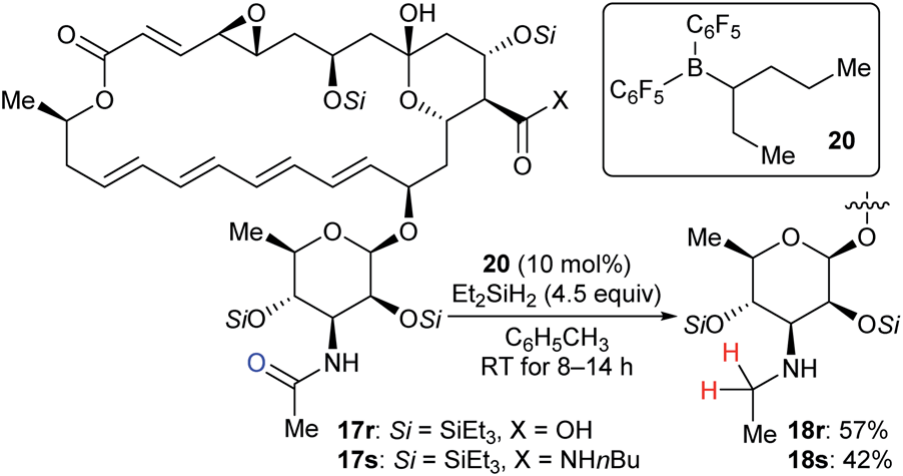
Selective deoxygenation of mycosamine acetamides catalysed by B(C_6_F_5_)_2_(hex-3-yl) (**20**) with Et_2_SiH_2_ as the reductant.

Based on the successful application of three boron catalysts with modified steric and electronic profiles for the selective modifications of natamycin, Gagné and co-workers developed the mixed alkyl(fluoroaryl)borane catalyst B(C_6_F_5_)_2_(hex-3-yl) (**20**), which is generated *in situ* by the hydroboration of hex-3-ene with Piers' borane HB(C_6_F_5_)_2_, for the chemoselective reduction of mycosamine acetamides (Scheme 20).^32*a*^ The reaction of acetamide derivatives of natamycin **17r** and **17s** with Et_2_SiH_2_ (4.5 equiv.) in the presence of **20** (10 mol%) led to the selective reduction of the *N*-acetamide to the *N*-ethyl mycosamine derivatives of natamycin **18r** and **18s** in useful yields without the competing reduction of other sites. Later, these authors developed a heteroleptic borane catalyst B(C_6_F_5_)_2_(CH_2_CH_2_CH_2_Bpin) for the mild reduction of tertiary alkyl amides, *N*-acetyl proline dipeptides, and even cyclosporine A with Me_2_EtSiH or Et_2_SiH_2_ as reductants with good functional-group tolerance (not shown).^47^

**Scheme 21:**
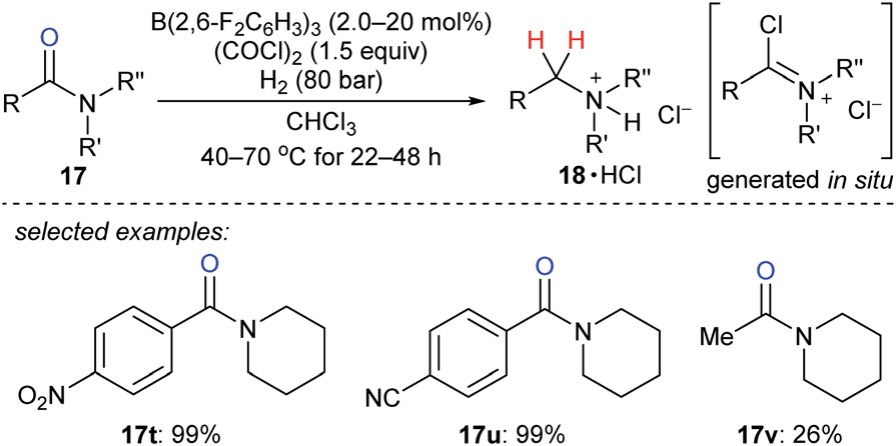
Deoxygenation of tertiary amides catalysed by B(2,6-F_2_C_6_H_3_)_3_ with H_2_ as the reductant.

The reduction of tertiary amides with H_2_ as the reductant with the aid of oxalyl chloride as an activating agent catalysed by B(2,6-F_2_C_6_H_3_)_3_ was disclosed by Paradies, Grimme, and co-workers (Scheme 21).^48^ The process involves the *in situ* formation of a chloroiminium chloride intermediate by the reaction of the amide with oxalyl chloride and exhibits high functional-group tolerance towards ester, ether, nitro, cyano, or thiophenyl groups. The corresponding amines were isolated as their HCl salts. The reduction of acetylamide **17v** resulted in a low yield, and the authors attributed this to the polymerisation of the corresponding chloroiminium chloride intermediate. Mechanistic investigations indicated the key role of chloride as an active Lewis base in borane-mediated H_2_ activation.

**Scheme 22:**
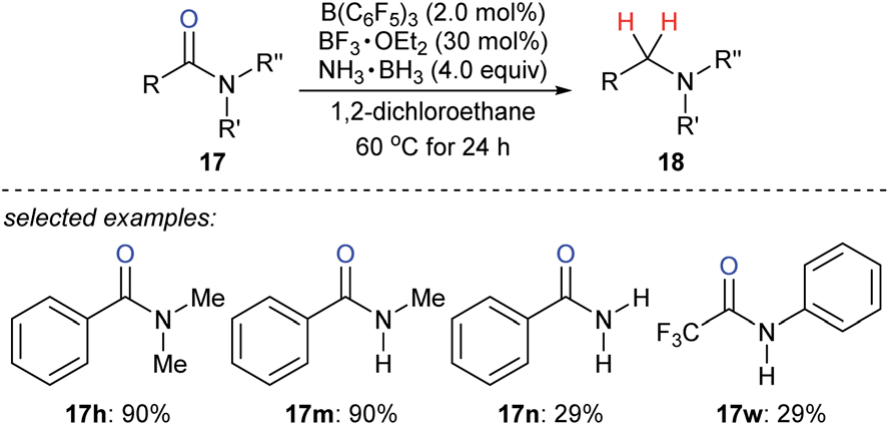
Deoxygenation of amides catalysed by B(C_6_F_5_)_3_ with NH_3_·BH_3_ as the reductant.

Ammonia borane is an ideal H_2_ storage material^49^ owing to its high storage capacity (19.6 weight% H), low molecular weight (30.87 g mol^−1^), good stability against air and moisture, easy availability, and simple handling. It has been intensively investigated as a reductant for the reduction of unsaturated C–C and carbon–heteroatom bonds.^50^ Xu, Fan, Xiao, and co-workers reported the reduction of various amides with ammonia borane as the reductant in the presence of catalytic amounts of B(C_6_F_5_)_3_ and BF_3_·OEt_2_ to provide a wide range of structurally diverse amines in good to excellent yields under mild reaction conditions with high functional-group tolerance (Scheme 22).^51^ The role of the BF_3_·OEt_2_ co-catalyst is to activate the carbonyl group of amide by Lewis adduct formation.

**Scheme 23:**
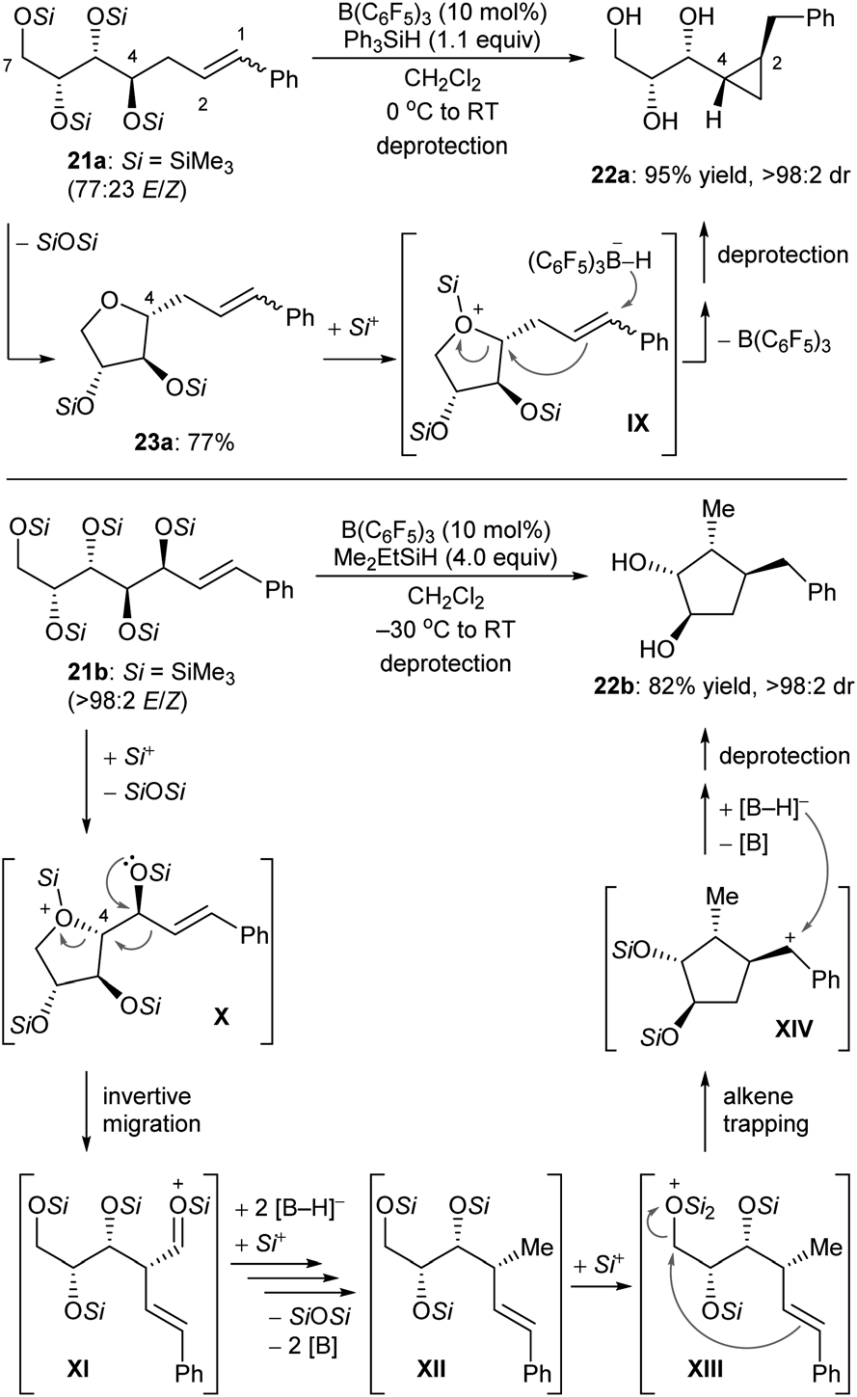
Carbocyclisation of unsaturated carbohydrates catalysed by B(C_6_F_5_)_3_ with Ph_3_SiH or Me_2_EtSiH as the reductant.

Some deoxygenation reactions accompanied by ring closure and rearrangement processes have been explored. This further highlights the synthetic applicability of defunctionalisation with B(C_6_F_5_)_3_/hydrosilane combinations. In 2016, Gagné and co-workers disclosed a B(C_6_F_5_)_3_-catalysed reductive carbocyclisation of unsaturated carbohydrates with hydrosilanes to give cyclopropanes and cyclopentanes with high efficiency and excellent diastereoselectivity (Scheme 23).^52^ The reaction of *gluco*-derived **21a** with Ph_3_SiH (1.1 equiv.) in the presence of B(C_6_F_5_)_3_ (10 mol%) generated a single diastereomer of cyclopropane **22a** in 95% yield (Scheme 23, top). This process involves B(C_6_F_5_)_3_-catalysed intramolecular nucleophilic substitution of the activated primary C7 position by C4–OSi of **21a** to form **23a**, which captures a “silylium ion” by the more basic cyclic oxygen atom to generate silyloxonium **IX**. Borohydride reduction of alkene moiety in **IX** induces a cyclisation event to yield cyclopropane **22a** after deprotection. Conversely, the allylic polyol derivative **21b** provided a single cyclopentane diastereomer **22b** in 82% yield under similar conditions (Scheme 23, bottom). After the formation of silyloxonium **X**, a 1,2-migration of the styryl group with inversion at C4 produces an silyloxycarbenium/silylcarboxonium ion intermediate **XI**, which is reduced by borohydride to the observed intermediate **XII**. Subsequent silylation of the primary silyl ether group of **XII** is followed by cyclisation through nucleophilic attack of the alkene to the activated C7 carbon atom. This generates a benzylic cation which is further reduced by borohydride to give cyclopentane **22b** after deprotection.

**Scheme 24:**
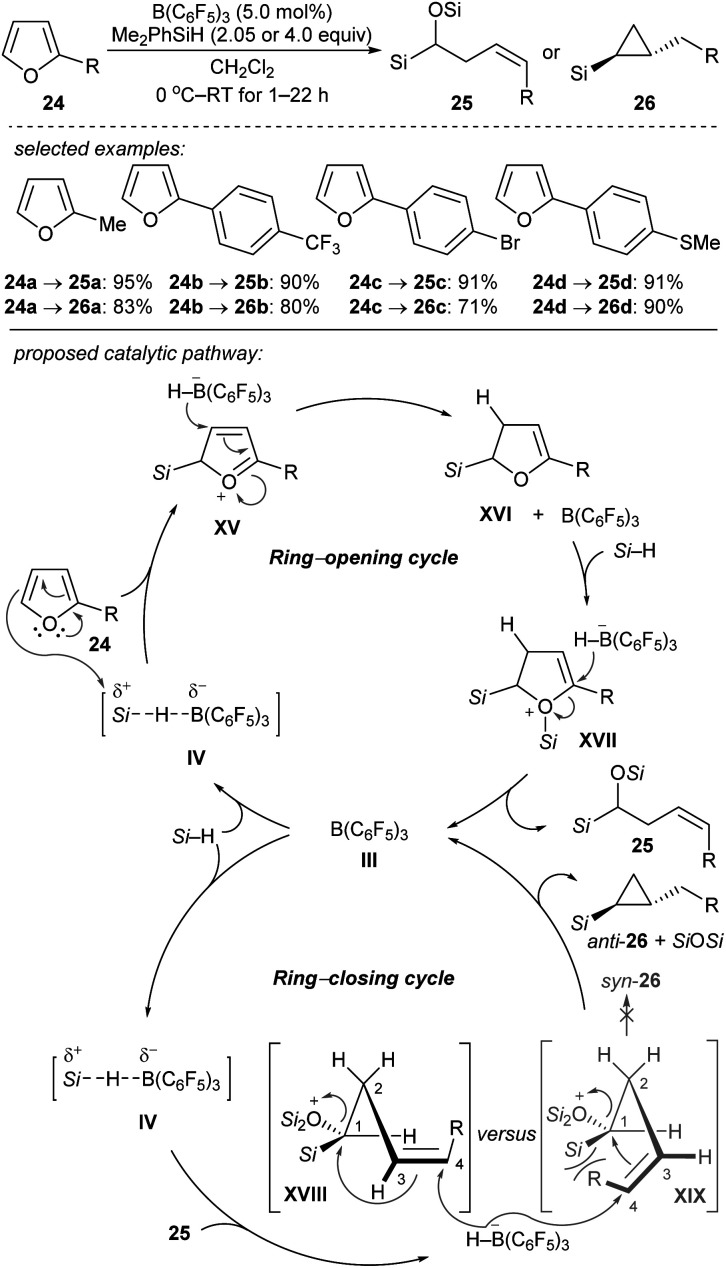
Ring-opening and -closing cascades of furans catalysed by B(C_6_F_5_)_3_ with Me_2_PhSiH as the reductant.

Chang and co-workers reported the stereocontrolled conversion of furans into *Z*-configured homoallylic silanes **25** and *anti*-substituted cyclopropyl silanes **26** through selective ring-opening and subsequent ring-closing processes (Scheme 24).^53^ The B(C_6_F_5_)_3_-catalysed double hydrosilylation of furans with Me_2_PhSiH (2.05 equiv.) afforded **25** in excellent yields and with high stereoselectivity (Scheme 24, top). The subsequent cyclopropanation can be simply achieved by the addition of further equivalents of the hydrosilane to furnish **26** with exclusive *trans*-selectivity. Isolation of ring-opened intermediates is not required. The authors proposed a cascade of B(C_6_F_5_)_3_-catalysed ring-opening (by two-fold hydrosilylation) and ring-closing reactions (by intramolecular cyclopropanation) (Scheme 24, bottom). The selective borohydride attack at the α-carbon of intermediate **XVII** and at the C4 of silyloxonium species **XVIII** leads to *trans*-(2-alkyl)cyclopropyl silanes exclusively. Later, these authors further reported the B(C_6_F_5_)_3_-catalysed reductive carbocyclisation of homoallylic alcohols and dihydro-2*H*-pyrans with Me_2_EtSiH or PhSiH_3_ as reductants to construct a range of 1,2-disubstituted (hetero)arylcyclobutanes under mild reaction conditions with high efficiency and excellent *cis*-selectivity.^54^ Mechanistic studies suggested a stepwise, dual ring-closing pathway (not shown).

**Scheme 25:**
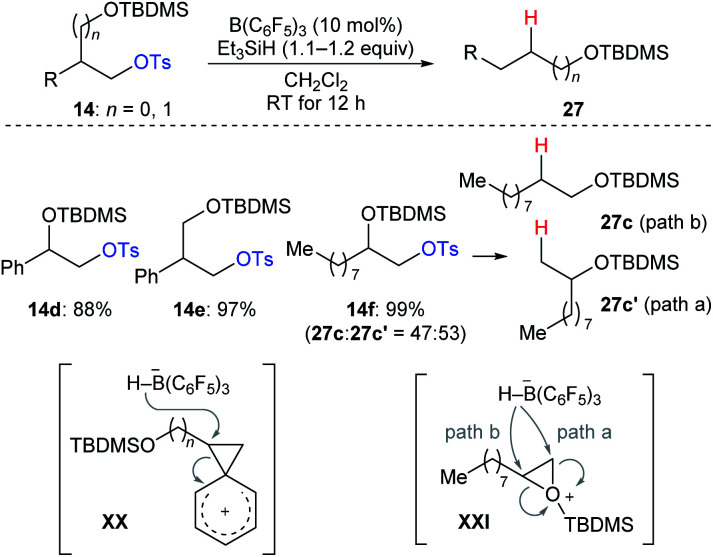
Rearrangement of primary alkyl tosylates catalysed by B(C_6_F_5_)_3_ with Et_3_SiH as the reductant.

During their investigation of chemoselective deoxygenation of protected 1,*n*-diols, Oestreich and co-workers found that diols **14d–f** were partially or fully transformed into the rearranged products (Scheme 25).^35^ The authors proposed that these processes involve phenonium ion intermediates **XX** for substrates **14d** and **14e** with anchimeric assistance by an adjacent aryl group or a three-membered silyloxonium ion intermediate **XXI** for aliphatic **14f**. A similar rearrangement was also observed by Song and co-workers (not shown).^36^

**Scheme 26:**
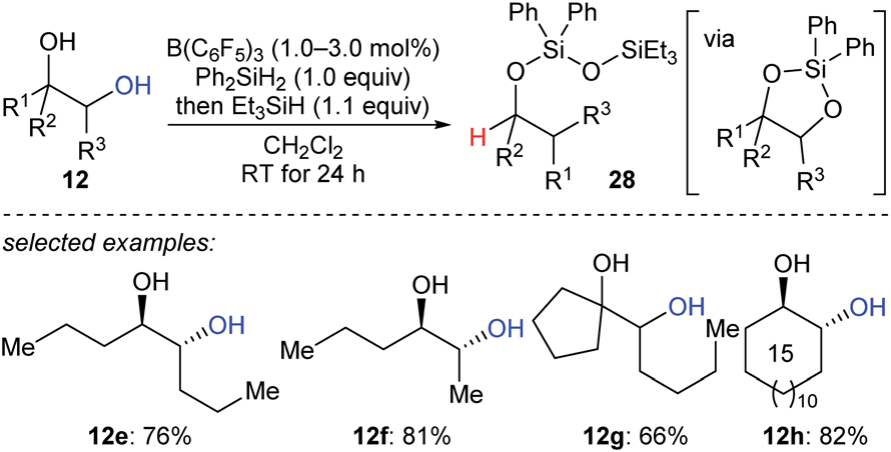
Pinacol-type rearrangement of 1,2-diols catalysed by B(C_6_F_5_)_3_ with Et_3_SiH as the reductant.

A reductive pinacol-type rearrangement of internal 1,2-diols was described by Morandi and co-workers (Scheme 26).^55^ By employing a combination of B(C_6_F_5_)_3_ and two hydrosilanes, a broad range of structurally diverse 1,2-diols **12e–h** underwent reductive rearrangement with inversion to give primary and secondary alcohols. This process involves the formation of a cyclic siloxane, and mechanistic investigations indicated that alkyl migration occurs prior to deoxygenation in internal diols due to the hyperconjugative and steric effects of the alkyl substituent.

## Boron Lewis acids-catalysed decarbonylation

**Scheme 27:**
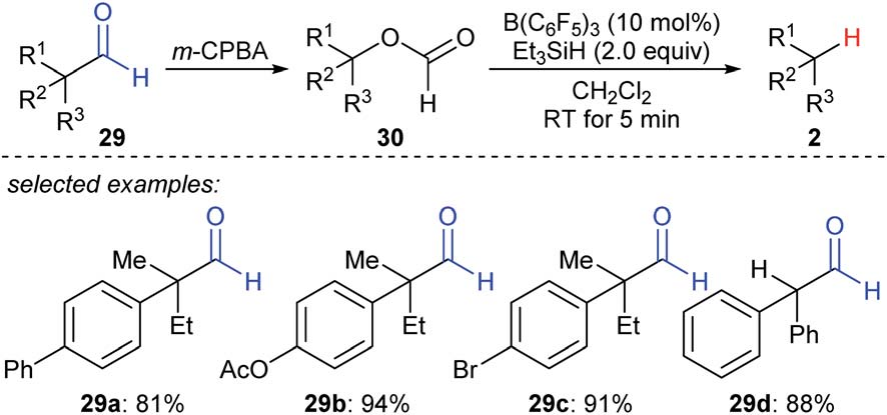
Formal decarbonylation of α-branched aliphatic aldehydes catalysed by B(C_6_F_5_)_3_ with Et_3_SiH as the reductant.

A formal decarbonylation of aliphatic aldehydes **29a–d***via* a sequence of Baeyer–Villiger oxidation and B(C_6_F_5_)_3_- or BF_3_·OEt_2_-catalysed deoxygenation of the resulting formate with Et_3_SiH as the reductant was developed by Richter and Oestreich (Scheme 27).^56^ Mechanistic investigations suggested that an S_N_1 mechanism is involved for the deoxygenation process.

## Boron Lewis acids-catalysed desulfurisation

**Scheme 28:**
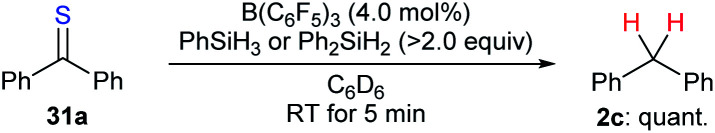
Desulfurisation of thiobenzophenone catalysed by B(C_6_F_5_)_3_ with PhSiH_3_ or Ph_2_SiH_2_ as reductants.

The combination of a Lewis acid catalyst and a hydrosilane has been widely used for the activation of C–O bonds in the above deoxygenation processes. However, application of this catalytic system to the cleavage of other carbon–heteroatom bonds is far less explored. During their investigation of B(C_6_F_5_)_3_-catalysed chemoselective postpolymerisation modification of poly(phenylsilane), Rosenberg and co-workers observed the formation of diphenylmethane as a result of overreduction of thiobenzophenone (**31a**).^57^ The authors demonstrated that the desulfurisation of **31a** with PhSiH_3_ or Ph_2_SiH_2_ as reductants occurred rapidly to furnish diphenylmethane (**2c**) in quantitative yield (Scheme 28).

**Scheme 29:**
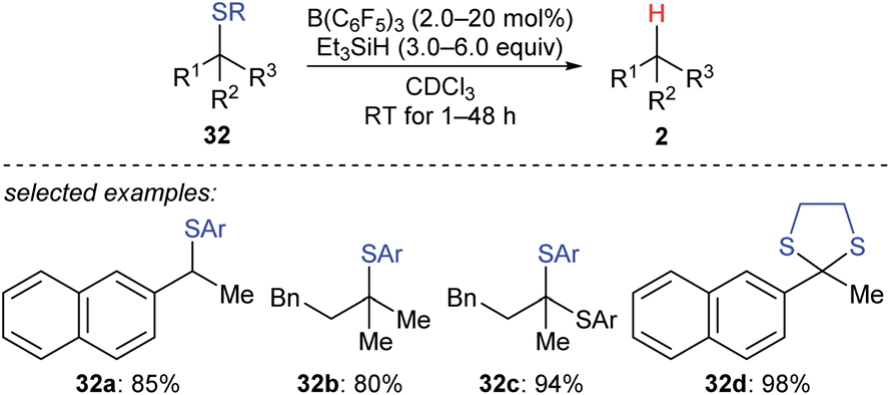
Desulfurisation of sulfides catalysed by B(C_6_F_5_)_3_ with Et_3_SiH as the reductant. Ar = *p*-ClC_6_H_4_.

A detailed investigation of B(C_6_F_5_)_3_-catalysed desulfurisation of various sulfides **32a–d** was disclosed by Akiyama and co-workers (Scheme 29).^58^ The desulfurisation of various benzyl and alkyl sulfides and dithianes with Et_3_SiH as the reductant in the presence of a catalytic amount of B(C_6_F_5_)_3_ generated the corresponding hydrocarbons in good yields under mild reaction conditions with high chemoselectivity. This process could be applied to the deprotection of dithioacetals.

## Boron Lewis acids-catalysed deamination

**Scheme 30:**
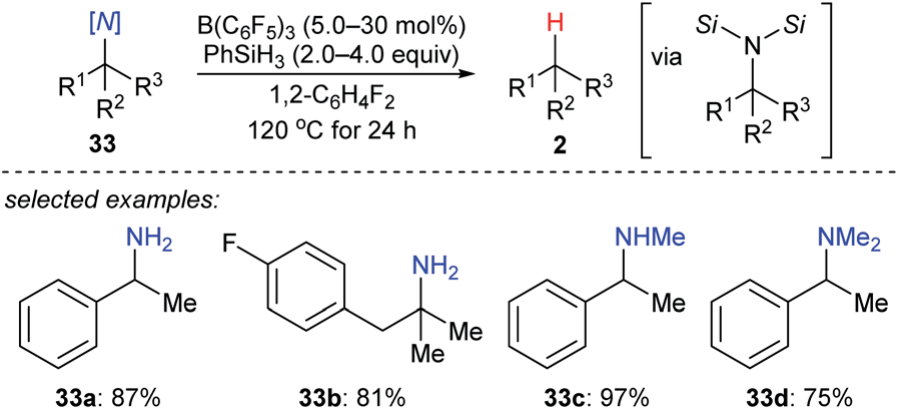
Deamination of amines catalysed by B(C_6_F_5_)_3_ with PhSiH_3_ as the reductant.

More recently, the utility of boron Lewis acid/hydrosilane combinations in the cleavage of C–N bonds to effect catalytic deamination was described by Fang and Oestreich (Scheme 30).^59^ With B(C_6_F_5_)_3_ as the catalyst and PhSiH_3_ as the reductant, a broad range of 1°, 2°, and 3° amines **33a–d** as well as heterocumulenes (not shown) was converted into the corresponding hydrocarbons at 120 °C. Yields were moderate to good. The relative reactivity of 1°, 2°, and 3° benzylic amines under catalytic conditions was investigated and was opposite to the order of reactivity seen in the deoxygenation of C–O bonds. This process involves the formation of bissilylammonium borohydride intermediates. These dissociate into the corresponding benzylic carbocations which could be further captured by the borohydride to generate the defunctionalised products.

## Boron Lewis acids-catalysed dehalogenation

**Scheme 31:**
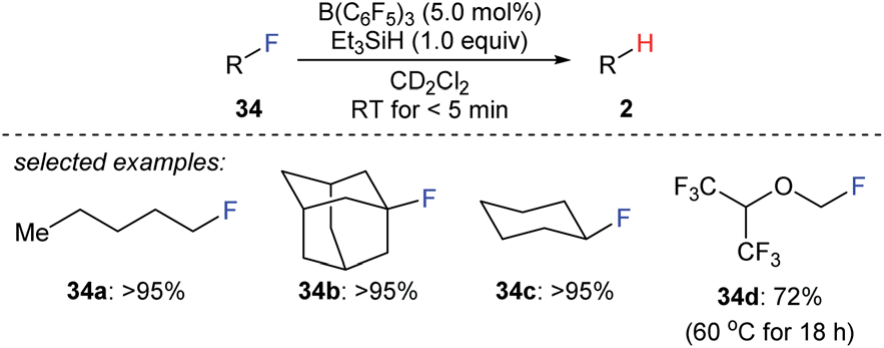
Hydrodefluorination of alkyl fluorides catalysed by B(C_6_F_5_)_3_ with Et_3_SiH as the reductant.

The combined use of boron Lewis acid and hydrosilanes can also be employed to the cleavage of carbon–halogen bonds. In 2012, Caputo and Stephan reported a mild B(C_6_F_5_)_3_-catalysed hydrodefluorination of 1°, 2°, and 3° alkyl fluorides with Et_3_SiH as the reductant to afford the corresponding hydrocarbons in good to excellent yields (Scheme 31).^60^ The hydrodefluorination of 1,1,1,3,3,3-hexafluoro-2-(fluoromethoxy)propane (**34d**) was more sluggish, and a temperature of 60 °C was required at which the trifluoromethyl groups remained intact. The authors attributed the slower reaction to the presence of the ethereal oxygen rendering the C–F bond less polar, thus leading to a weak donor–acceptor interaction between the substrate and the boron Lewis acid. Later, the selective hydrodefluorination of a C1-fluorinated glucose derivative with TMDS as the reductant catalysed by Piers' borane, (C_6_F_5_)_2_BH, generated *in situ* from (C_6_F_5_)_2_BOH was described by Zhang, Park, and Chang (not shown).^61^

**Scheme 32:**
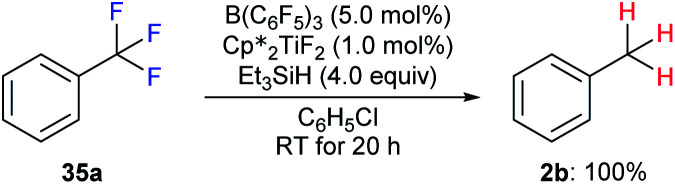
Hydrodefluorination of trifluorotoluenes catalysed by the combination of B(C_6_F_5_)_3_ and 
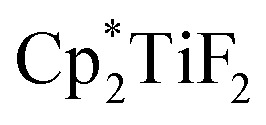
 with Et_3_SiH as the reductant.

The hydrodefluorination of trifluorotoluenes with Et_3_SiH as the reductant catalysed by B(C_6_F_5_)_3_ alone was unsuccessful. By adding an extra group 4 metal complex as a co-catalyst, Lamač and co-workers realised this transformation.^62^ Among the metallocene co-catalysts screened, 
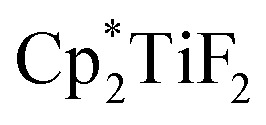
 was the most active co-catalyst and also promoted the hydrodechlorination of the aliphatic halogenated solvent, CHCl_3_. A quantitative yield of toluene was obtained for the hydrodefluorination of trifluorotoluene (**35a**) with Et_3_SiH catalysed by the combination of B(C_6_F_5_)_3_ and 
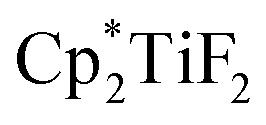
 in PhCl (Scheme 32). Et_3_SiF was formed as a by-product, and a higher selectivity was achieved compared to previously reported catalytic systems by suppressing Friedel–Crafts side products generated by the alkylation of toluene with substrate.

**Scheme 33:**
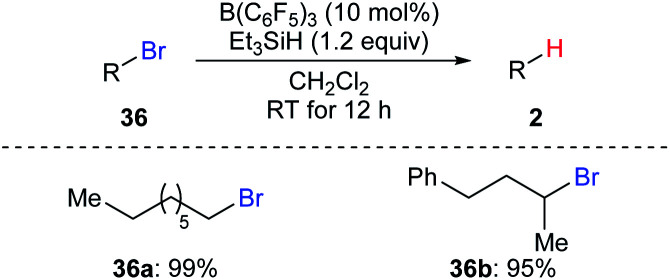
Hydrodebromination of alkyl bromides catalysed by B(C_6_F_5_)_3_ with Et_3_SiH as the reductant.

Oestreich and co-workers also demonstrated B(C_6_F_5_)_3_-catalysed hydrodebromination of primary and secondary alkyl bromides **36a** and **36b** with Et_3_SiH as the reductant at room temperature (Scheme 33).^35^ However, hydrodechlorination of the corresponding alkyl chloride using this catalytic system was unsuccessful.

## Summary and outlook

During the past two decades, significant achievements have been made in the field of defunctionalisation on the basis of boron Lewis acid catalysis, which exhibits comparable or even superior catalytic activity and selectivity to transition metal catalysis. Starting from Piers's seminal discovery of B(C_6_F_5_)_3_-catalysed hydrosilylation and Gevorgyan's early works on B(C_6_F_5_)_3_-catalysed deoxygenation, numerous reductive alcohol deoxygenations by combinations of boron Lewis acids and hydride sources have been developed. Especially Gagné showcased the impressive chemo-, regio-, and stereoselectivity that can be achieved with this tool. In addition, this boron Lewis acid catalysis has been successfully extended to the cleavage of C–S, C–N, and carbon–halogen bonds. However, the efficiency and selectivity of boron Lewis acid catalysis still needs to be further improved. Functional-group tolerance remains an issue. Thus, the development of air- and moisture-stable and easy-to-prepare and -handle catalysts with high activity and selectivity is desirable. Next to the significant advances made in the area of deoxygenation, selective decarbonylation, desulfurisation, deamination, and dehalogenation have just begun to flourish.

## Conflicts of interest

There are no conflicts to declare.
